# The Insurance Bill and Voluntary Hospitals

**Published:** 1911-10-21

**Authors:** J. Courtney Buchanan

**Affiliations:** Secretary and House Governor. Metropolitan Hospital. Kingsland Road, N.E.


*To the Editor of* The Hospital.

### THE INSURANCE BILL AND VOLUNTARY HOSPITALS.

*To the Editor of* The Hospital.

Sir,—May I first of all express my sincere appreciation of your kindly references to me in the current and preceding numbers of The Hospital; but my purpose in writing to you is to ask for certain information which, I believe, I can obtain from no other source. I am anxious to know, partly for my Committee's information (though I confess I have another object in view as well), the following, namely :—

1. The present cost of hospitals in London, the provinces, Scotland, and Ireland. It was stated at Manchester that "hospitals require in this country from ten to fifteen millions a year"; but I venture to suggest that there is a very large gap between ten and fifteen millions, which you probably can easily lessen.

I notice also on page 11 of your exceedingly interesting and instructive article on "Co-operation and Intercommunication" in The Hospital that you give the percentages of hospitals' income which is more or less secured. What is the approximate figure of income which is not secured? I cannot follow you in your suggestion of the way to arrive at it (page 11 top); and this is the figure, of course, which will be affected by operation of the Insurance Bill.

2. What would be the effect on the hospitals' income if they were granted state aid? What amount (if any), in your opinion, would hospitals lose if they were in receipt of state aid?

3. What amount would probably be required from the State in payment for treatment of persons at present treated at the hospitals, who will be "insured persons"? It seems to me that the answer to this question can only be the expression of a personal opinion, because the estimates of the number of insured persons who, after the enactment of the Insurance Bill, will be treated in the hospitals is so uncertain.

I feel very sorry indeed if I am asking you for figures which it will be difficult to obtain, but I was advised to apply to you and I should be very grateful if you could let me have an answer to this letter.—Yours faithfully,

Kingsland Road, N.E. J. Courtney Buchanan,

October 9. Secretary and House Governor.

Metropolitan Hospital.

[We have dealt with the questions raised by Mr. Buchanan in the article on National Insurance, Voluntary and State Hospitals, on p. 71.—Ed., The Hospital.]

